# Association of HbA1C and comfort with diabetes self-management among adolescents and young adults with type 1 diabetes

**DOI:** 10.3389/fcdhc.2024.1304577

**Published:** 2024-05-10

**Authors:** Obichi Onwukwe, Erika L. Lundgrin

**Affiliations:** ^1^ Case Western Reserve University School of Medicine, Cleveland, OH, United States; ^2^ Department of Pediatric Endocrinology, University Hospitals Rainbow Babies and Children's, Cleveland, OH, United States

**Keywords:** adolescent, young adult, type 1 diabetes mellitus, glycated hemoglobin, self report, sociodemographic factors, regression analysis, transition to adult care

## Abstract

**Introduction:**

Adolescents and young adults (AYA) living with type 1 diabetes (T1D) are a vulnerable demographic at risk for sub-optimal glycemic outcomes at a time when they are taking over their diabetes management. The purposes of this study were to examine levels of self-reported comfort with diabetes management tasks among AYA living with T1D and to describe the relationships among comfort levels, sociodemographic factors, and HbA1c.

**Methods:**

During a routine diabetes care visit, AYA aged 15–23 years old living with T1D received a transition survey to self-assess their comfort level with different diabetesmanagement tasks.

**Results:**

Among 161 participants who completed the survey (median age 17 years, median diabetes duration 7 years, 82.3% White, 40.9% female, 66.5% with private insurance, and median HbA1c 8.8%), comfort with diabetes management tasks was generally rated highly (median overall comfort level of 4.5 out of 5), irrespective of race or insurance type. Regression analysis revealed that higher self-reported comfort level with diabetes management tasks was associated with a higher HbA1c (p = 0.006), after controlling for age, sex, race, insurance type, and diabetes duration.

**Discussion:**

These findings suggest that self-reported comfort with independently managing T1D may not be a sufficient metric in assessing AYA patients’ need for further intervention to optimize glycemic outcomes as they transition from pediatric to adult diabetes care, and highlights the importance of continuity of care to support diabetes management during this transitional period.

## 1 Introduction

Emerging adulthood is a critical stage in patient development. Wood et al. (1) describe this period as an opportunity for healthcare providers to influence their patients’ life course health development. It is also a vulnerable time period during which patients’ diabetes management and control may deteriorate, increasing the risk of diabetes complications. Data from the Diabetes Control and Complications Trial (DCCT) suggest that the mean HbA1c for adolescents was 1%–2% higher than that of adults (2). Similarly, a 2019 analysis of the Type 1 Diabetes (T1D) Exchange Registry found that, between 2016 and 2018, mean HbA1c in youth aged 15–18 living with T1D was higher than both their younger and older counterparts. This study also highlighted that the American Diabetes Association (ADA) glycemic targets are not being met by most individuals living with T1D, and that HbA1c levels are especially high in adolescents and young adults (AYA), compared to children and older adults. Moreover, studies have indicated that young adults are at increased risk for diabetes-related complications in their late 20s (3). However, to date, research is limited on comfort with diabetes self-management and readiness to transition to adult care among older youth or emerging adults living with T1D.

Existing literature has also identified other factors, such as socioeconomic status and race, influencing health outcomes in patients living with T1D. Secrest et al. (4) examined adults aged 24–32 living with T1D and found lack of college degree, low income level, and non-professional occupation to be associated with complications related to diabetes, such as renal disease, coronary artery disease, and autonomic neuropathy. Among youth less than 22 years of age, higher socioeconomic status has also been shown to be associated with lower HbA1c levels (5). Furthermore, Chalew et al.’s (6) retrospective analysis of HbA1c in a pediatric population revealed that patients identifying as Black had higher HbA1c and mean blood glucose than their counterparts who identified as White. Additionally, Kahkoska et al.’s (7) analysis of the data from the SEARCH for Diabetes in Youth study concluded that there are indeed racial and ethnic disparities in long-term glycemic control. The results from the 2018 analysis indicated that Hispanic youth and non-Hispanic Black youth were more likely to be in a higher HbA1c trajectory group, representing a moderate baseline and a major increase in HbA1c, than their non-Hispanic White counterparts.

This retrospective, cross-sectional study utilized a transition survey that was provided to AYA living with T1D to examine levels of self-reported comfort with diabetes self-management tasks and investigate their relationship with glycemic outcomes and sociodemographic factors.

## 2 Methods

**Figure 1:**
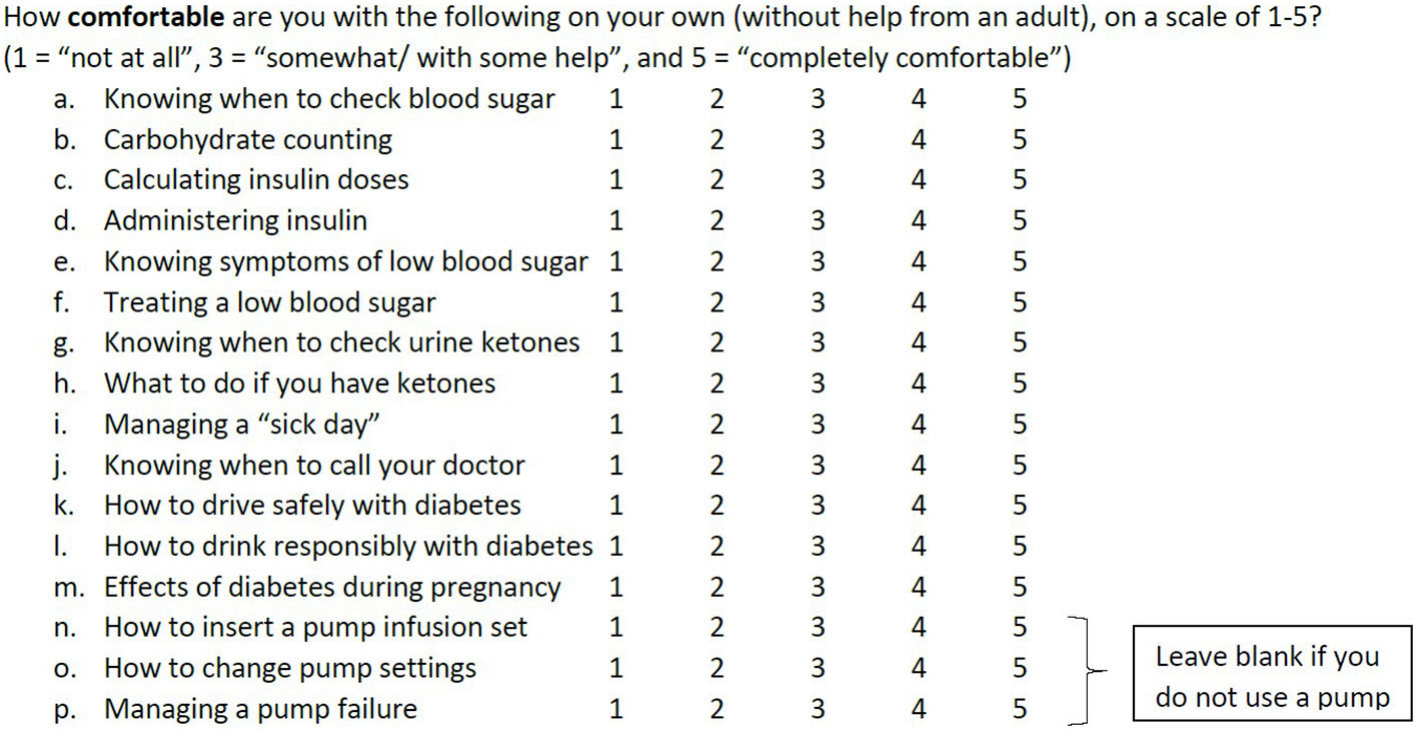
Transition survey question regarding comfort with diabetes self-management tasks.

As part of a routine pediatric endocrinology clinic visit in 2018, an optional transition survey was provided to AYA aged 15 years and older who had been diagnosed with T1D for at least 3 months. The brief “Type 1 Diabetes Transition Survey for Teens and Young Adults” utilized a five-point Likert scale to assess level of comfort with 16 diabetes self-management tasks with a goal of targeting education provided at the clinic visit (**Figure 1**). Some survey questions reflected routine T1D management tasks, such as knowing when to check blood glucose and calculating insulin dose. Other survey questions involved diabetes knowledge relevant to AYA, such as understanding the impact of diabetes on driving and pregnancy. Completed surveys were scanned into the electronic medical record (EMR) after the visit.

Approval from the Institutional Review Board (IRB) was obtained for retrospective chart review and cross-sectional analysis of sociodemographics, glycemic outcomes, and self-reported comfort with diabetes self-management tasks, including all patients who had completed a transition survey between 10 August and 4 December 2018. HbA1c levels at the time of survey completion, transition survey answers, as well as age, sex, race/ethnicity, insurance type, and diabetes duration were obtained through retrospective chart review of the EMR.

Age, diabetes duration, HbA1c, and self-reported comfort levels were described using median and interquartile range. Each patient’s average comfort with all tasks was calculated, and the population average comfort level of the cohort was also described using median and interquartile ranges. After chart review, the insurance type was classified as either private or non-private. Regression analysis was used to assess the impact of self-reported comfort level with diabetes self-management tasks and preparedness to manage diabetes, independently on HbA1c, after controlling for age, sex, race, insurance type, and diabetes duration. Comparisons of survey measures among different sociodemographic groups (e.g., race and insurance type) were also analyzed using regression analysis. Statistical analysis was completed using JMP Pro 16.

## 3 Results

A total of 161 AYA with T1D (40.9% female participants; median age, 17) completed the transition survey. Of this group, 82.3% self-identified as White and 17.7% self-identified as non-White. The majority of participants (*n* = 107, 66.5%) had private insurance, but this was much less common among non-White participants (only 35.5% with private insurance). The median average comfort level reported by participants was 4.46 (out of 5), which was similar among White and non-White participants (**Table 1**). The median HbA1c of the entire group was 8.8%, and only approximately 5% of participants had an HbA1c meeting the ADA-recommended goal of <7%. When stratified by race, White participants have a slightly lower median HbA1c of 8.5%, compared to non-White race participants, who had an elevated median HbA1c of 10.9%.

**Table 1:** Demographic and glycemic data and average comfort level, stratified by race.

Characteristic	All AYA (*n* = 161)	White (*n* = 130)	Non-White (*n* = 31)
Age (years)	17 (16–19)	17 (16–19)	17 (16–18)
Sex (female)	66 (40.9%)	50 (38.5%)	16 (51.6%)
Diabetes duration	7 (5–11)	7 (5–11)	8 (4–11)
Insurance type (private)	107 (66.5%)	96 (73.8%)	11 (35.5%)
HbA1c (%)	8.8 (7.7–10.7)	8.5 (7.7–10.1)	10.9 (8.5–13.2)
Average comfort	4.46 (3.96–4.85)	4.45 (3.98–4.86)	4.46 (3.81–4.84)

Data presented as either *n* (%) or median (interquartile range).

**Figure 2:**
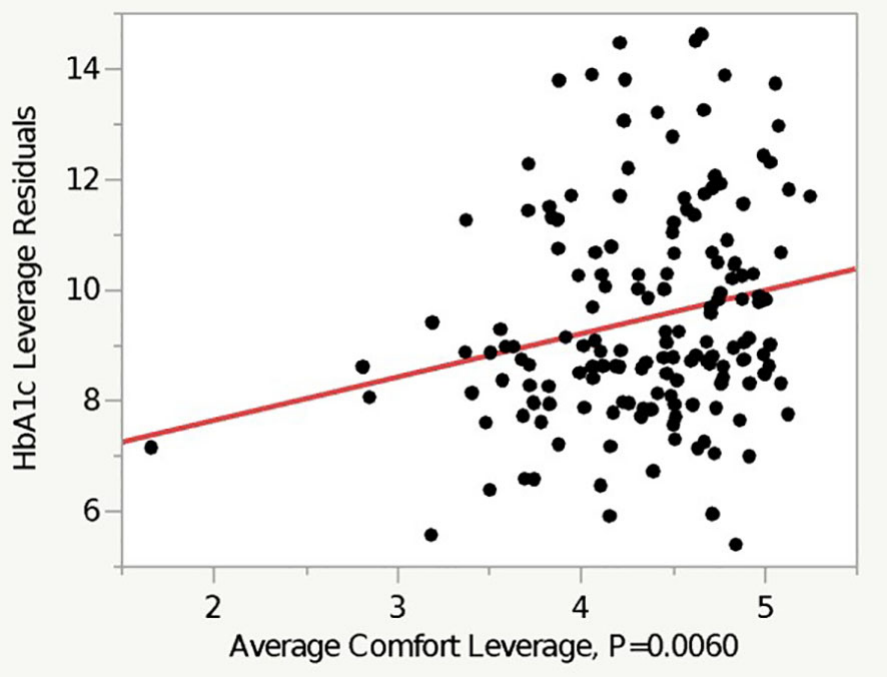
Residuals vs. leverage plot of the relationship between average comfort and HbA1c.

Regression analysis revealed that higher self-reported comfort level with diabetes management tasks was associated with a higher HbA1c (*p* = 0.006), after controlling for age, sex, race, insurance type, and diabetes duration (**Table 2** and **Figure 2**). On average, White participants had an HbA1c level that is 0.89% lower than their non-White counterparts (*p* < 0.0001), after controlling for other factors. Private insurance and female sex were both similarly significantly associated with a lower HbA1c in the multivariate model (**Table 2**).

**Table 2:** Multiple linear regression model predicting HbA1c.

Variable	Coefficient (*β*)	*p*-value
Age (years)	0.06	0.4636
Sex (female)	−0.50	0.0045*
Diabetes duration (years)	0.07	0.0598
Insurance type (private)	−0.39	0.0356*
Race (white)	−0.89	<.0001*
Average comfort	0.79	0.0060*

* denote the p-values that are statistically significant.

## 4 Discussion

Our study shows that, overall, AYA report high levels of comfort with independently managing T1D. Despite this high level of comfort, most AYA (95%) were not meeting HbA1c goals as outlined by the ADA. These findings are consistent with existing literature exploring transition of care preparation among this demographic. When using the Readiness of Emerging Adults with Diabetes Diagnosed in Youth (READDY) tool among AYA, Kamoun et al. (8) similarly found that patients reported high levels of overall confidence. Additionally, data from the T1D Exchange Clinic Network registry show poorer glycemic outcomes in young adults when compared to other age groups (9). However, while various studies have analyzed glycemic outcomes among AYA living with T1D, research is limited regarding the correlation between comfort with diabetes self-management and preparedness to transition to adult diabetes care, and glycemic outcomes. Interestingly, we found that patients who reported higher levels of comfort with independently managing T1D tended to have worse glycemic outcomes, as evidenced by higher HbA1c. These results suggest that self-reported comfort with independently managing T1D may not be a sufficient metric in assessing AYA patients’ need for further intervention to optimize glycemic outcomes as they transition from pediatric to adult providers. It is important to note that transition readiness surveys, including the one utilized in this study and the READDY tool, encompass knowledge that can be demonstrated. The findings of this study highlight a potential role for a more immersive educational experience that challenges patients to demonstrate and apply their perceived knowledge and mastery of diabetes management skills within the learning environment. Additionally, these results highlight the need for ongoing support to enhance diabetes self-management during the transition from pediatric to adult diabetes care.

Upon examination of sociodemographic trends in the present study, we found racial and socioeconomic disparities in glycemic outcomes similar to prior research. In particular, our data showed that patients who identified as White had lower HbA1c when compared to their non-White counterparts (coefficient −0.89, *p* < 0.0001). Likewise, using insurance type as a surrogate for socioeconomic status, patients with private insurance also had lower HbA1c levels (coefficient −0.39, *p* = 0.0256). Findings by Agarwal et al. (10) suggest that non-Hispanic Black and Hispanic young adults report lower use of diabetes technology, higher diabetes distress, and lower self-management (10). While we controlled for sociodemographic factors in our study, we did not examine other social determinants of health, such as access to transportation services and diabetes technology, as well as health literacy of caretakers/guardians. It is probable that the racial and socioeconomic trends noted in our research and those of others may be due to one or more social determinants of health. In addition to racial and socioeconomic disparities, our study found disparities among different sexes. Female patients had statistically significantly better glycemic outcomes, as evidenced by lower HbA1c, than their non-female counterparts (coefficient −0.5, *p* = 0.0045). This finding contributes to the currently limited breadth of literature on gender- and sex-specific differences in glycemic outcomes in AYA. In 2018, Maiorino et al. (11) found that adult women with T1D in Italy had higher HbA1c levels than men, while Shah et al. (12) found no gender differences among adult men and women in the United States. Given the limited sample size (*n* = 161) of the present study, further research is needed to further examine and explain possible differences in glycemic outcomes of AYA versus adult women.

A further limitation of our study is that we did not utilize an established, validated transition survey. The transition survey administered to participants was an inter-departmental survey, designed to be concise in order to facilitate efficient data collection during a routine clinic visit. Additionally, there was a predominance of patients with private insurance (*n* = 107) in the study population. Despite these limitations, this study is among the first to analyze the association between diabetes self-management comfort and glycemic outcomes in the AYA demographic. Further studies would be helpful to affirm findings in a larger and diverse sample of AYA. Finally, participants of this study were not queried to self-identify barriers they faced in performing various knowledge components. Additional research is needed to clarify the effect of other social determinants of health on the relationship between diabetes self-management comfort and glycemic outcomes in AYA, and should address possible barriers to optimizing glycemic outcomes in this population.

## Data availability statement

The raw data supporting the conclusions of this article will be made available by the authors, without undue reservation.

## Ethics statement

The studies involving humans were approved by University Hospitals Sparta Institutional Review Board. The studies were conducted in accordance with the local legislation and institutional requirements. The ethics committee/institutional review board waived the requirement of written informed consent for participation from the participants or the participants’ legal guardians/next of kin because the study was a retrospective chart review and analysis of data that was initially obtained for a quality improvement project.

## Author contributions

OO: Formal analysis, Writing – original draft, Writing – review & editing. EL: Conceptualization, Writing – review & editing.

## References

[1] Wood D Crapnell T Lau L Bennett A Lotstein D Ferris M. Emerging adulthood as a critical stage in the life course. In: Halfon N Forrest CB Lerner RM Faustman EM, eds. Handbook of Life Course Health Development. Cham (CH): Springer; November 21, 2017. 123-143. doi: 10.1007/978-3-319-47143-3_7 31314293

[2] Maahs DM West NA Lawrence JM Mayer-Davis EJ. Epidemiology of type 1 diabetes. Endocrinol. Metab. Clinics. (2010) 39:481–97. doi: 10.1016/j.ecl.2010.05.011 PMC2925303 20723815

[3] Bryden KS Dunger DB Mayou RA Peveler RC Neil HAW. Poor prognosis of young adults with type 1 diabetes: a longitudinal study. Diabetes Care. (2003) 26:1052–7. doi: 10.2337/diacare.26.4.1052 12663572

[4] Secrest AM Costacou T Gutelius B Miller RG Songer TJ Orchard TJ. Associations between socioeconomic status and major complications in type 1 diabetes: the pittsburgh epidemiology of diabetes complication (edc) study. Ann. Epidemiol. (2011) 21:374–81. doi: 10.1016/j.annepidem.2011.02.007 PMC3079455 21458731

[5] Galler A Lindau M Ernert A Thalemann R Raile K. Associations between media consumption habits, physical activity, socioeconomic status, and glycemic control in children, adolescents, and young adults with type 1 diabetes. Diabetes Care. (2011) 34:2356–9. doi: 10.2337/dc11-0838 PMC3198300 21926289

[6] Chalew S Kamps J Jurgen B Gomez R Hempe J. The relationship of glycemic control, insulin dose, and race with hypoglycemia in youth with type 1 diabetes. J. Diabetes Complications. (2020) 34:107519. doi: 10.1016/j.jdiacomp.2019.107519 32303406 PMC8978593

[7] Kahkoska AR Shay CM Crandell J Dabelea D Imperatore G Lawrence JM. Association of race and ethnicity with glycemic control and hemoglobin a1c levels in youth with type 1 diabetes. JAMA Netw Open. (2018) 1:e181851–e181851. doi: 10.1001/jamanetworkopen.2018.1851 30370425 PMC6203341

[8] Kamoun C Khoury JC Beal SJ Crimmins N Corathers SD. Opportunities for enhanced transition of care preparation for adolescents and emerging adults with type 1 diabetes: use of the readdy transition tool. Diabetes Spectr. (2022) 35:57–65. doi: 10.2337/ds20-0104 35308159 PMC8914586

[9] Miller KM Foster NC Beck RW Bergenstal RM DuBose SN DiMeglio LA. Current state of type 1 diabetes treatment in the us: updated data from the t1d exchange clinic registry. Diabetes Care. (2015) 38:971–8. doi: 10.2337/dc15-0078 25998289

[10] Agarwal S Kanapka LG Raymond JK Walker A Gerard-Gonzalez A Kruger D. Racial-ethnic inequity in young adults with type 1 diabetes. J. Clin. Endocrinol. Metab. (2020) 105:e2960–9. doi: 10.1210/clinem/dgaa236 PMC7457963 32382736

[11] Maiorino MI Bellastella G Casciano O Petrizzo M Gicchino M Caputo M. Gender-differences in glycemic control and diabetes related factors in young adults with type 1 diabetes: results from the metro study. Endocrine. (2018) 61:240–7. doi: 10.1007/s12020-018-1549-9 29455365

[12] Shah VN Wu M Polsky S Snell-Bergeon JK Sherr JL Cengiz E. Gender differences in diabetes self-care in adults with type 1 diabetes: findings from the t1d exchange clinic registry. J. Diabetes Complications. (2018) 32:961–5. doi: 10.1016/j.jdiacomp.2018.08.009 30121205

